# Sustained-release drug delivery systems

**DOI:** 10.1038/s41433-024-03134-w

**Published:** 2024-05-17

**Authors:** Rachel Williams, Helen Cauldbeck, Victoria Kearns

**Affiliations:** 1 Department of Eye and Vision Science, University of Liverpool, Liverpool, L69 3BX UK; 2 Department of Chemistry, University of Liverpool, Liverpool, L69 3BX UK

**Keywords:** Outcomes research, Eye diseases

## Abstract

The design and development of a sustained-release drug delivery system targeting the administration of active pharmaceutical ingredients (APIs) to the eye could overcome the limitations of topically administered eye drops. Understanding how to modify or design new materials with specific functional properties that promote the attachment and release of specific drugs over longer time periods, alongside understanding clinical needs, can lead to new strategic opportunities to improve treatment options. In this paper we discuss two approaches to the design or modification of materials to produce a sustained therapeutic effect. Firstly, we discuss how the synthesis of a peptide hydrogel from a naturally-derived antimicrobial material led to the design of a bandage contact lens which may be able to be used prophylactically to reduce post-surgery infection. Secondly, we discuss how silicone oil tamponade agents used to treat retinal detachments can have adjunctive behaviour to enhance the solubility of the anti-proliferative drug retinoic acid and produce a sustained release over several weeks. These studies are the result of close partnerships between clinical ophthalmologists, materials scientists, and chemists, and illustrate how these partnerships can lead to comprehensive understandings that have the potential to change patient outcomes.

## Abstract (zh)

设计和开发针对眼部有效药物成分(APIs)给药的缓释药物递送系统, 可克服局部滴眼液的局限性。了解如何修饰或设计具有特定功能特性的新材料, 以促进特定药物在更长时间段内的附着和释放, 并且满足临床需求, 可带来新的改进治疗方案的战略机遇。本文讨论了两种设计或改进的材料以产生持续治疗效果的方法。首先, 我们讨论了如何用天然抗菌材料合成的多肽水凝胶设计绷带镜, 这种绷带镜可预防性地用于减少术后感染。其次, 我们讨论了用于治疗视网膜脱离的硅油填充剂如何具有辅助作用, 以增强抗增殖药物维甲酸的溶解性, 并在数周内持续释放。这些成果得益于临床眼科医生、材料学家和化学家之间的密切合作, 文中阐明了如何加深理解这些合作关系以改变患者的临床预后。

## Introduction

The majority of active pharmaceutical ingredients (APIs) are administered to the eye by topical eye drops. It is well known, however, that this is an inefficient method of achieving therapeutic levels of the API in the relevant tissues [1]. Eye drops can be difficult for patients to administer themselves, and a large volume of the drop rapidly exits the eye either out of the eye down the face, or through the lacrimal duct. There are also anatomical and physiological barriers to the APIs entering into the ocular tissues and particularly reaching the back of the eye.

To achieve a more effective distribution of APIs into the anterior ocular tissues, the goal must be to enhance the permeability of the tissues to the APIs and increase the length of time the APIs are in contact with those tissues; there have been significant advances in topical formulations to achieve this [1]. The most effective way to achieve therapeutic levels of APIs in the posterior ocular tissues is to administer them directly into the posterior segment. Frequent injection into this part of the eye, however, has many disadvantages so the goal must be to provide controlled and sustained levels of APIs over time from each injection. There are opportunities to use our understanding of the materials science of synthetic materials that are already used in ocular applications, or new materials with appropriate properties, to tailor sustained drug delivery systems to enhance treatments. The appropriate solution, however, will be specific to the API, the target tissue, and the treatment regime required (Table 1).

**Table 1:** Examples of drug delivery systems and their advantages and limitations for (a) anterior and posterior segments and (b) formulation type.

a		
Drug delivery system	Advantages	Limitations
Anterior segment		
Eye drops	Non-invasive	Low bioavailability
Ocular inserts (solid)	• Local drug release • Potential drug release over several months	• Very careful control and understanding of polymer vehicle, drug formulation and excipient •Invasive
Contact lenses	• Different methods available to incorporate drugs in the lenses • Increased bioavailability • Non-invasive	• Changes to oxygen permeability of lenses • Storage issues of the lenses with drug incorporated
Posterior segment		
Eye drops	• Non-invasive	• Conventionally poor bioavailability, but recent advances using nanomedicines and other approaches have reached clinical trial • Poor patient compliance • Repetitive dosing regimen
Intravitreal injection	• Enhanced bioavailability	• Invasive • Requires repeat administration • Risk of complications
Subretinal/suprachoroidal delivery	• Reduced concentration of drug required • Delivery to anatomically-closed space • Precise delivery to target tissue	• Invasive and more technically challenging • Usually requires vitrectomy • Risk of complications
Intravitreal implants (degradable/non-degradable)	• Enhanced bioavailability • Sustained delivery so less frequent delivery	• Tendency for non-linear drug release, with lower doses at longer times post-implantation
Tamponade agents	• Exploiting the presence of a device that’s already being implanted into vitreous cavity	• Difficult to get sufficient drug incorporated/dissolved into tamponade • Sustained release often difficult to achieve • Limited indications for use of tamponade agent • Need for surgical removal of tamponade agent

b		
Formulation type	Advantages	Limitations
Suspensions	• Increased retention compared to eye drops	• Ocular irritation • Poor stability • Blurred vision
Emulsions (micro/nano)	• Increased retention compared to eye drops • Increased stability compared to suspensions • Increased permeability	• Blurred vision • Ocular irritation
Gels (semi-solid)	• Increased retention compared to eye drops • Increased stability compared to suspensions • Increased permeability	• Blurred vision • Ocular irritation
Liposomes	• Relatively easy preparation • Increases retention compared to eye drops	• Composition very important particularly around charge • Issues of sterilisation
Polymeric micro/nano-particles	• High surface to volume ratio • Drugs can be attached to surface or encapsulated • Good penetration (particularly for nano-particles)	• Limitation to drug loading • Lack of uniformity of particle dispersion • Storage stability
Nano-fibres	• High surface to volume ratio • Adjustable mechanical and degradation profiles	• Requires careful polymer choice and manufacturing process
Dendrimers	• Branched structure can lead to high drug loading capacity • Selective targeting of tissues can reduce off-target toxicity	• Careful consideration of the cytotoxicity of the dendrimer vehicle is required
Microneedles	• Minimally invasive • Controlled sustained drug delivery	• Material properties still need optimisation • Clinical/patient use requires training

For drug delivery to the ocular surface, the easiest approach is to increase the residence time of the API in contact with the tissues. Many studies have aimed to achieve this by increasing the viscosity of the formulation *via* the production of ointments, emulsions, and gels [1]. There are also opportunities to use viscosity enhancers to increase the solubility of the API and thus increase its bioavailability to the ocular tissues. An alternative, well-researched approach has been to use contact lenses as a drug delivery system [2, 3]. Hydrophilic contact lenses can simply trap topically administered APIs in the pre-existing tear film layer between the lens and the corneal surface, enhancing residence of the API at the surface. Alternatively, the contact lens can be modified to be used as a reservoir for specific APIs, providing an enhanced release profile of the API over time. Many studies have involved commercially available contact lenses and the simple absorption of APIs from solution into their structure with the release of the APIs being controlled by diffusion. There are opportunities, however, to incorporate APIs in other forms, for example, as particles, films, emulsions, and liposomes, to modify their solubility, and thus bioavailability, and the loading concentration and release rate of the API. Optimisation of the drug delivery needs to consider the material properties of the contact lens, the chemistry of the API, and the size, formulation, and distribution of the API within the contact lens to achieve the appropriate therapeutic dose.

Topical delivery methods used to administer APIs to the posterior ocular tissues which use any of the methods discussed above to enhance bioavailability and residence time will help to increase the transport of these APIs through either the transvitreal route *via* the aqueous fluid, the uveal/scleral route through drainage through the Schlemm’s canal or the periocular route through the conjunctival vasculature [4, 5]. The levels of API reaching the required posterior tissues are, however, severely limited by many physiological barriers. It is generally well-recognised that direct intravitreal delivery is the most effective route of administration for these treatments, although not without its disadvantages. A wide range of studies have reported on approaches to enhance the control and sustained delivery of APIs by modifying the formulation of the API into, for example, micro- or nano-particles, emulsions, liposomes, or solid degradable or non-degradable implants. In each of these cases, there are opportunities to use our understanding of the material science of the API/formulation interactions with the biological environment to optimise the therapeutic delivery to the desired tissues.

This paper will review two specific approaches we have taken to achieve a sustained therapeutic effect. Firstly, the delivery of an antimicrobial effect to the front of the eye using a novel peptide hydrogel contact lens and, secondly, the delivery of anti-inflammatory and antiproliferative effects to the posterior segment through the modification of silicone oil tamponades.

## Poly-ε-lysine bandage contact lenses

Poly-ε-lysine (peK) is a peptide composed of around 25–30 lysine amino acids with the peptide bond between the carboxylic acid on the central carbon and the amine group on the end of the side chain (ε-amine group). This creates a linear positively charged peptide with the α-amine groups free along the backbone of the peptide giving the peptide antimicrobial properties and as such it has been used as a food preservative for many years. Hydrogels can be synthesised from poly-ε-lysine using di-carboxylic acids to cross-link the peptides using an *N*-hydroxysuccinimide (NHS)/1-ethyl-3-(3-dimethylaminopropyl) carbodiimide (EDCI)-mediated technique.

A family of hydrogels with different properties can be synthesised by varying the density of the polymer, the length of the cross-linking di-carboxylic acid (i.e. the number of repeat alkyl groups between the two carboxylic acid groups), and the cross-link density (i.e. the proportion of the α-amine groups involved in the cross-linking). We have shown that a peptide hydrogel can be synthesised with sufficient mechanical properties, transparency, and water content to be a potential bandage contact lens and can be cast in a contact lens mould using a polymer density of 0.071 gcm^–3^, octanedioic acid (C8) as the cross-linker with a 60 mole% cross-link density [6]. This hydrogel had an elastic modulus of 0.6 MPa, a refractive index of 1.390, and a water content of around 70% all of which are in the same region as commercially available hydrogel contact lenses.

**Fig. 1:**
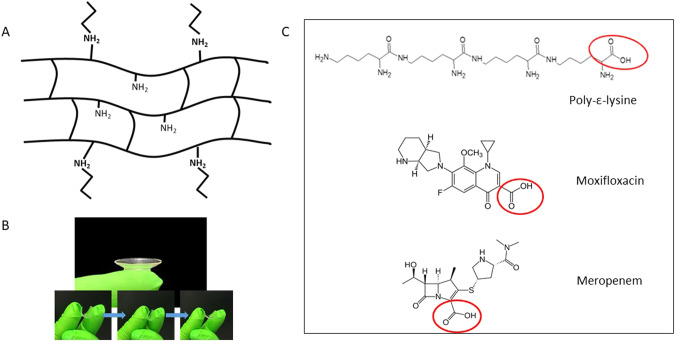
Poly-ε-lysine bandage contact lens. **A** Schematic of the hydrogel structure with potential API binding sites; **B** Cast lens and demonstration of pinching flexibility; **C** Potential APIs that can be bound *via* carboxyl group.

The antimicrobial properties of poly-ε-lysine are a result of its poly-cationic character and its ability to disrupt the bacterial cell walls. When cross-linked, however, there may not be a long enough chain of the positively charged α-amine groups to achieve this. In the 60% cross-linked hydrogel, we have 40% free amine groups which can be used to covalently bind further free poly-ε-lysine peptides anchored by their carboxylic acid end group leaving the positively charged amine groups to interact with the bacterial cell walls (Fig. 1). Alternatively, antibiotics, which contain carboxylic acid groups, such as moxifloxacin and meropenem, can be electrostatically bound to the hydrogel.

Bandage contact lenses are often used following cornea surgery but currently available bandage contact lenses do not have any antimicrobial properties. The design of a bandage contact lens with inherent antimicrobial material properties has the potential to reduce the incidence of post-surgery infection. In the longer term, the ability to optimise the electrostatic attachment of negatively charged antibiotics to the positively charged peptide hydrogel could lead to more sustained release of the antibiotics to overcome the need for such frequent administration of eye drops as is required currently. We have evaluated the antimicrobial properties of this peptide hydrogel against *Staphylococcus aureus, Escherichia coli* [6], *Pseudomonas aeruginosa* [7, 8], *Candida albicans* [9] and *Acanthamoeba castellanii* [10].

**Fig. 2:**
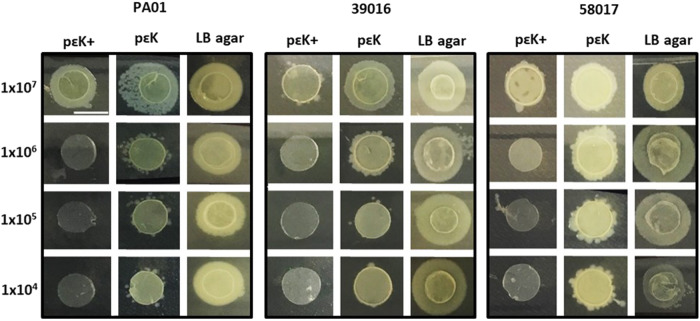
Reduction in *Pseudomonas aeruginosa* outgrowth from poly-ε-lysine hydrogels with pendent poly-ε-lysine (pɛK+), poly-ε-lysine hydrogels (pεK) and LB agar discs. The hydrogels and LB agar discs were incubated overnight in *Pseudomonas aeruginosa* (PAO1, PA39016, and PA58017) at 10^3^, 10^4^, 10^5^, 10^6^, and 10^7^ CFU for 24 h, removed from buffer, and plated onto LB agar plates, incubated overnight at 37 °C. Scale bar = 6 mm [7].

**Fig. 3:**
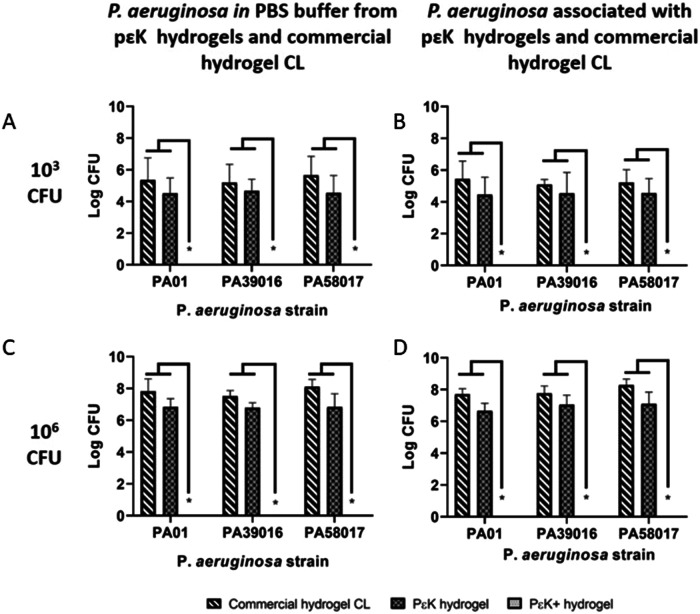
Antimicrobial activity of poly-ε-lysine hydrogels with pendent poly-ε-lysine (pɛK+), compared with poly-ε-lysine (pεK) hydrogels and commercial hydrogel CL against *Pseudomonas aeruginosa* isolates. Viable *Pseudomonas aeruginosa* in PBS buffer (**A**, **C**) and associated with pεK+ hydrogel (**B**, **D**), compared with pεK hydrogel and commercial hydrogel CL. PεK+ hydrogel, pεK hydrogel, and commercial hydrogel CLs were inoculated with *P aeruginosa* (PAO1, PA39016, and PA58017) at 10^3^ (**A**, **B**) and 10^6^ (**C**, **D**) CFU for 24 h. Viable bacterial counts were determined as CFU. Values represent mean of four independent experiments, error bars represent the standard deviation. *P < 0.05 using two-way ANOVA and post hoc Tukey’s analysis [7].

*P. aeruginosa* is one of the most common causes for microbial keratitis (MK). Using the laboratory strain of *P. aeruginosa* (PAO1) and two MK strains (PA39016, and PA58017) we showed that the poly-ε-lysine hydrogel with pendant attached poly-ε-lysine was able to reduce the outgrowth of the bacteria culture in comparison with the poly-ε-lysine hydrogel alone and an LB agar control (Fig. 2) [7]. In this study, the materials were incubated with the bacteria in culture with seeding densities up to 10^7^ colony forming units (CFU) for 24 h before being plated on to LB agar plates overnight. Only at the highest seeding density was there any evidence of bacterial outgrowth from the poly-ε-lysine hydrogel samples with the pendant poly-ε-lysine demonstrating a very high level of inhibition of cell attachment and survival on these hydrogels. In comparison, with the *P. aeruginosa* interaction with a commercially available hydrogel bandage contact lens (Filcon II 2, 77% water content, Ultravision, Leighton Buzzard, UK) at two seeding densities (10^3^ and 10^6^) we demonstrated a statistically significant reduction in the growth of the bacteria in contact with the poly-ε-lysine hydrogel with pendant poly-ε-lysine both when associated with the materials surfaces and in the media surrounding the materials (Fig. 3).

**Fig. 4:**
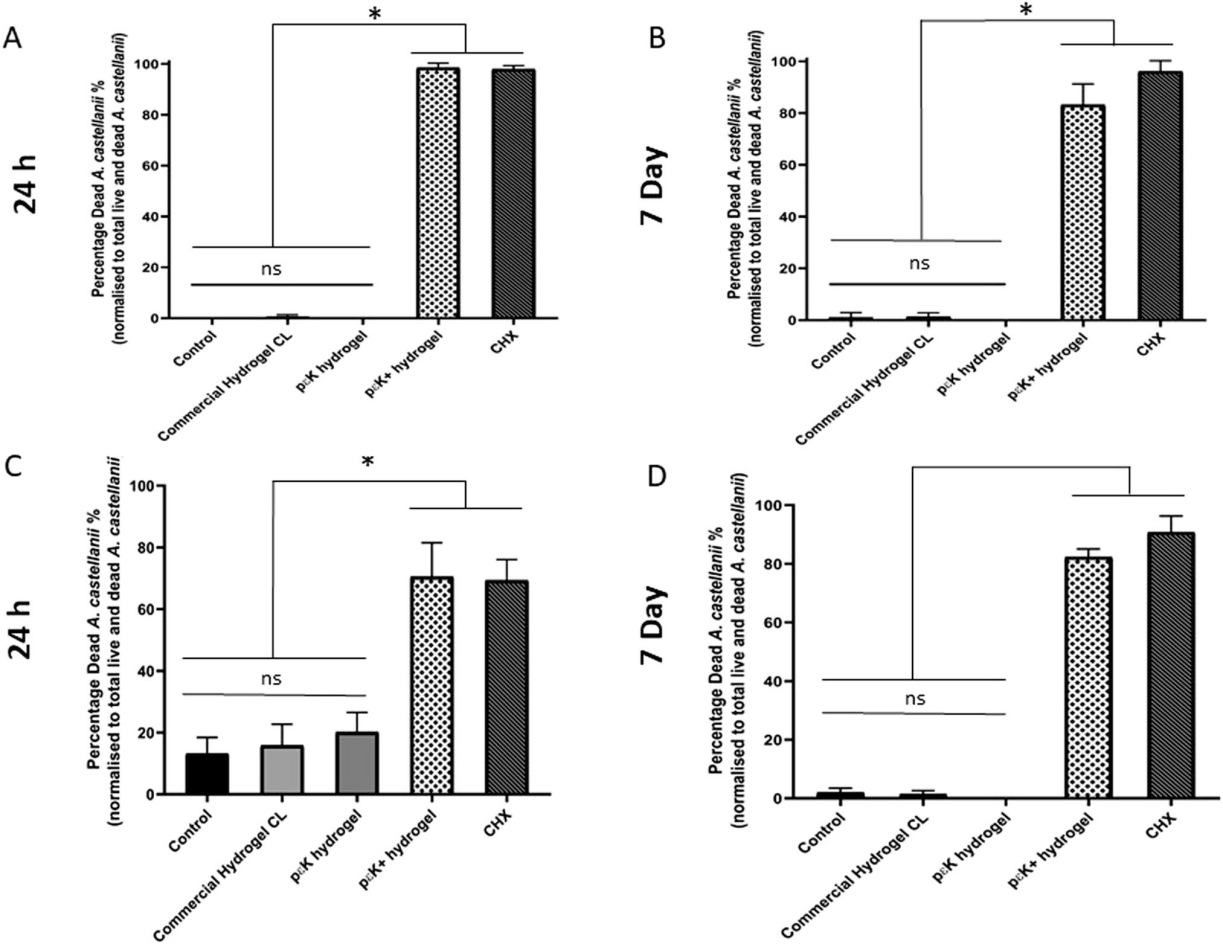
Toxicity of poly-ε-lysine hydrogels with pendent poly-ε-lysine (pɛK+), compared with poly-ε-lysine (pεK) hydrogels and commercial hydrogel CL against *Acanthamoeba castellanii.* **A** Graph to show the percentage of dead trophozoites at 24 h; **B** Graph shows the percentage of dead trophozoites at 7 days to total live and dead trophozoites; **C** Graph to show the percentage of dead cysts at 24 h; **D** Graph shows the percentage of dead cysts at 7 days to total live and dead trophozoites; Chlorohexidine was the positive control. Experiments were performed in triplicate (*n* = 3), with three wells per experiment and five fields of view in each well. One-way ANOVA was performed with a post hoc Tukey’s analysis, and *P* < 0.05 was considered significant [10].

Another difficult to treat infection often associated with wearing contact lenses is *Acanthamoeba* keratitis. Although rare it can cause severe loss of vision so opportunities to design contact lenses that could reduce the risk of this infection could have a significant benefit. We evaluated the effect of the poly-ε-lysine hydrogel with and without pendant poly-ε-lysine in comparison with the same commercially available hydrogel bandage contact lens and chlorohexidine standard treatment [10]. This study demonstrated that the poly-ε-lysine with pendant poly-ε-lysine caused significantly more death of the *Acanthamoeba* cultures in both the cyst and trophozoite form after incubation for 24 h and 7 days in comparison with poly-ε-lysine hydrogel alone, the commercially available hydrogel bandage contact lens and a tissue culture polystyrene control. The level of toxicity to the *Acanthamoeba* cultures of the poly-ε-lysine with pendant poly-ε-lysine was similar to that of chlorohexidine (Fig. 4).

These studies demonstrate the potential to design bandage contact lenses from an antimicrobial material that can be cross-linked to produce a hydrogel with appropriate physical and mechanical properties for this application. Using the peptide poly-ε-lysine in this application provides a large number of amine functional groups that can be used either to promote cross-linking and optimise the material properties or to bind further bioactive molecules and thus tailor the antimicrobial properties. The covalent attachment of pendant poly-ε-lysine to the contact lens promotes its interaction with the micro-organisms in culture which can inhibit their binding to the surface and under some conditions kill them. The positively charged nature of the peptide hydrogel, either with or without the pendant poly-ε-lysine, has the potential to be designed to optimise the attachment and release of negatively charged API molecules which may have a role in the treatment of MK. It should be noted that the regulatory approval of such systems is complicated by classification as a drug-device combination.

## Silicone oil tamponade agents

Silicone oil tamponades have been used successfully to treat complex retinal detachments for decades [11]. Their success relates to the chemical formula of the poly-dimethylsiloxane polymer leading to its hydrophobic character. This excludes aqueous and inflammatory mediators from the retinal tear and supports the retina against the underlying tissues while the tear heals. Modifications to silicone oils in recent years have generally involved changes to the molecular weight of the polymers to increase the viscosity of the tamponade and reduce emulsification [12–14] or the addition of semi-fluorinated alkanes to increase the tamponade specific gravity to improve treatment of inferior retinal detachments [15–17]. Proliferative vitreoretinopathy is a major complication of retinal detachment and there have been a number of studies [11] that have evaluated the potential of a silicone oil tamponade to deliver hydrophilic or hydrophobic anti-proliferative APIs [18, 19]. Poor solubility of API in the tamponade will lead to low, ineffective API loading, and uncontrolled release which potentially lead to toxicity due to very high levels of the API in the thin aqueous layer between the tamponade and the retina.

**Fig. 5:**
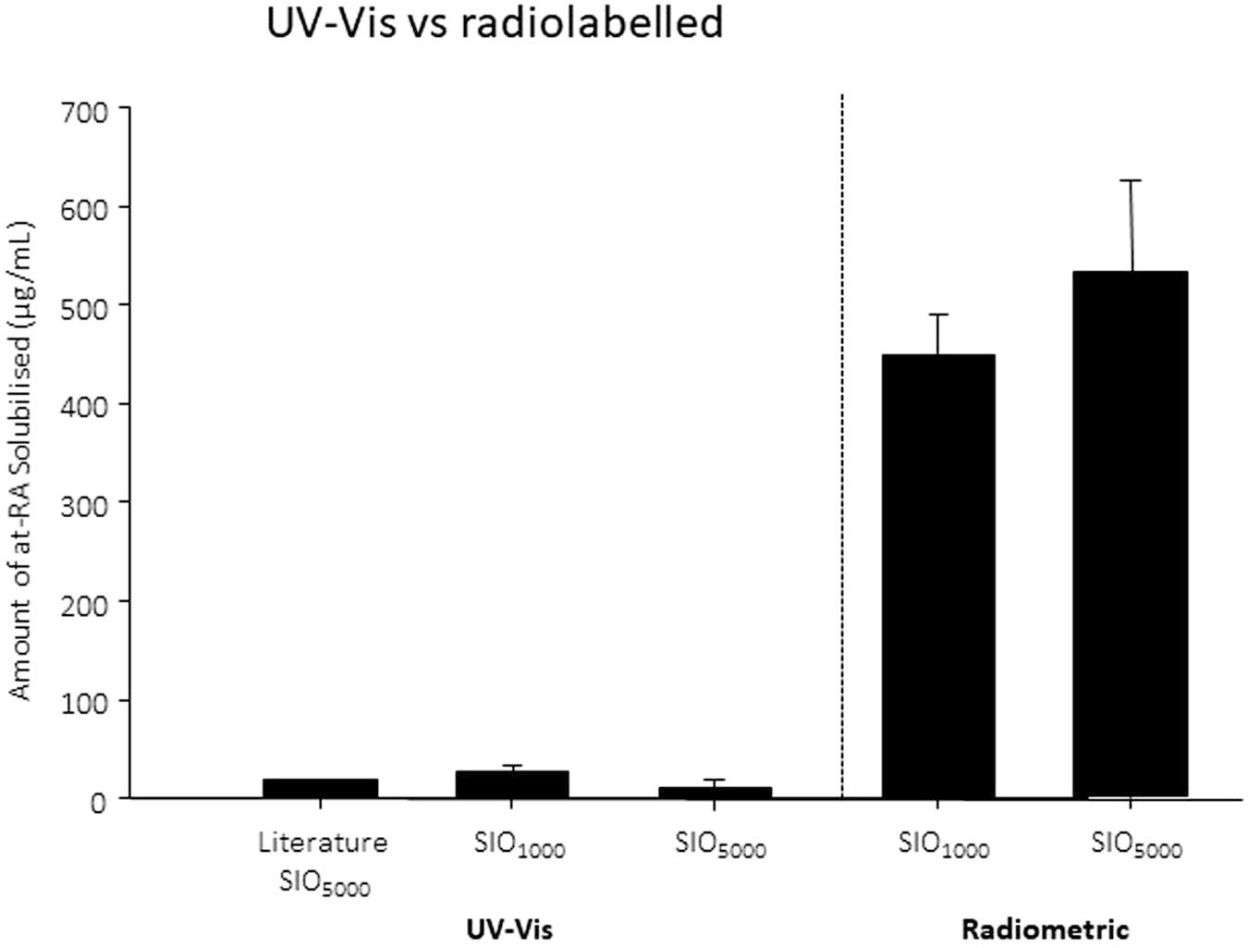
Comparison of saturation concentration of atRA measured in silicone oil (SIO) *via* acetone extraction followed by UV–Vis (SIO1000 *n* = 4, SIO5000 *n* = 3) and radioactivity measurements (SIO1000 *n* = 4; SIO5000 *n* = 4). Literature value taken from Araiz et al. [21] Error bars +1 standard deviation.

A third issue that we encountered was the accurate measurement of the amount of API in the silicone oil. This has typically been measured using UV–Vis spectroscopy which requires a number of extraction processes each of which can lead to loss of material and, in the case of all-trans retinoic acid (atRA), can be close to UV–Vis saturation in absorption. We directly compared the amount of atRA dissolved in silicone oil using UV–Vis and radiometric measurements with tritiated atRA; radiometric analysis demonstrated a 20-fold increase in the amount of API incorporated into the tamponade agent (Fig. 5) [19]. These results could have an inference on previous results on API loaded in silicone oil measured using UV–Vis spectroscopy.

**Fig. 6:**
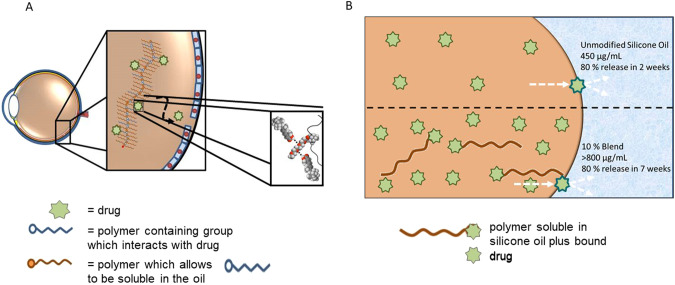
Schematic representation of silicone oil-API modification strategies to increase solubility and release. **A** API molecules co-dissolved within the silicone oil with graft co-polymers of methacrylated poly-dimethylsiloxane (PDMSMA) and methacrylated ethylene glycol (OEGMA) using RAFT polymerisation [18]; **B** Co-dissolved API conjugate between a short chain hydroxy-terminated poly-dimethylsiloxane and atRA *via* an esterification reaction.

To address the solubility of the API into the oil and then control of the release we took two different approaches with careful consideration of the chemistry of the silicone oil and the specific API (Fig. 6). In the first, we designed a short chain co-polymer that had functional molecules that would bind the API as well as functional molecules that would enhance solubility in the silicone oil [18]. In the second approach, we designed poly-dimethylsiloxane—API conjugates which were soluble in the silicone oil; we hypothesised this would increase the solubility and control the release of the free API [19].

**Fig. 7:**
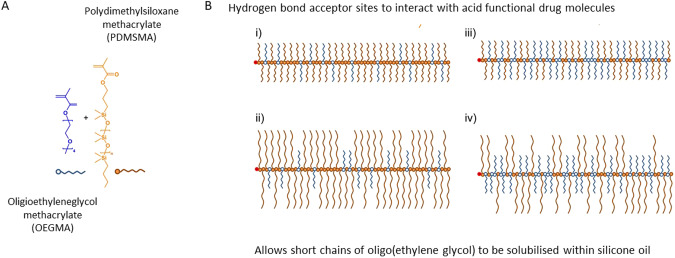
Graft copolymers with varying ratio, chain length and composition. **A** Monomethacryloxypropyl poly(dimethylsiloxane) methacrylate and oligo(ethylene glycol)monomethyl ethermethacrylate used during this study; **B** Schematic representation of structural and compositional variation within the statistical graft copolymers (**i**) low incorporation of hydrophilic grafts or similar chain length to hydrophobic chains; (**ii**) variation of hydrophobic graft length; (**iii**) increased composition of hydrophilic chains, and (**iv**) increased hydrophobic graft chain length at higher ratios of hydrophilic chains [18].

**Fig. 8:**
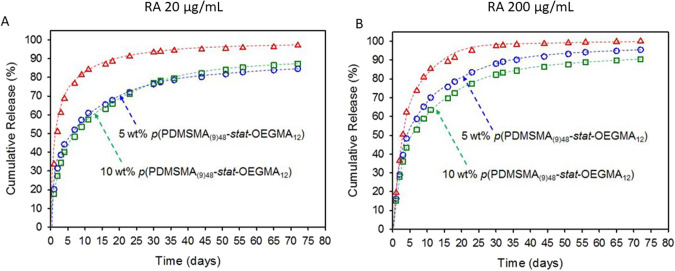
Studies of atRA release from silicone oil tamponades into aqueous media in the absence (open red triangles) and presence of statistical graft copolymer, using drug mixtures containing ^3^H-labelled atRA. Effect of increasing dissolved graft copolymer from 5 v/v% (open blue circles) to 10 v/v% (open green squares) within the silicone oil at all-trans retinoic acid concentrations of (**A**) 20 μg/mL, and (**B**) 200 μg/mL [18].

In the first approach, we designed and synthesised graft copolymers of methacrylated poly-dimethylsiloxane (PDMSMA) and methacrylated ethylene glycol (OEGMA) (Fig. 7) using RAFT polymerisation which would be added to the silicone oil tamponade. The PDMSMA promotes solubility in the silicone oil and the OEGMA promotes solubility of the hydrophilic API *via* hydrogen bonding. A number of different designs were synthesised with the understanding that the higher proportion and higher molecular weight of PDMSMA would lead to a greater solubility in silicone oil, whereas the greater the proportion of OEGMA would increase the solubility of the API. We found the optimal co-polymer design in relation to solubility was composed of 90 wt% of the shorter MW PDMSMA and 10 wt% OEGMA allowing 30 v/v% addition to silicone oil tamponade (Table 2). Adding this co-polymer to a silicone oil tamponade at 5 v/v% or 10 v/v% and dissolving atRA at either 20 µg/mL or 200 µg/mL we demonstrate that the co-polymer extended the release of the API in comparison to without the co-polymer. Specifically, we show that when atRA was added at 20 µg/mL the overall concentration of the co-polymer did not have an effect but adding the co-polymer extended the release of 80% of the API from 9 days to 40 days (Fig. 8). When 200 µg/mL of atRA was added we measured that it took 72 days for 90–95% of the API to be released. In this case, the level of co-polymer did influence the release rate with the higher level having a greater effect suggesting that there is a direct interaction between the co-polymer and API that influences the release rate. For example, at 20 µg/mL there was sufficient co-polymer to bind all the atRA whereas at 200 µg/mL, the 5% co-polymer was saturated with API and 10% co-polymer allowed a greater amount of API to bind. This demonstrated that design of the co-polymer could enhance API loading and the tailor the rate of release.

**Table 2:** Results of statistical graft homopolymer, copolymer, and terpolymer/silicone oil miscibility studies and target mole% of PDMS and ethylene glycol repeat units [18].

Target polymer	Target composition (mol% of total monomer)		Miscibility in silicone oil
Composition	EG	DMS	(% v/v)
*p*(OEGMA_60_)	100	0	<1
*p*(PDMSMA_(9)60_)	0	100	Miscible
*p*(PDMSMA_(57)60_)	0	100	Miscible
*p*(PDMSMA_(9)48_-*stat*-OEGMA_12_)	10	90	<30
*p*(PDMSMA_(9)30_-*stat*-OEGMA_30_)	30.8	69.2	<5
*p*(PDMSMA_(57)48_-*stat*-OEGMA_12_)	1.7	98.3	Miscible
*p*(PDMSMA_(57)30_-*stat*-OEGMA_30_)	6.5	93.5	Miscible

The second approach involved synthesising an API conjugate between a short chain hydroxy-terminated poly-dimethylsiloxane and the atRA *via* an esterification reaction (Fig. 6b) [19]. This API conjugate was added to a silicone oil tamponade and it was hypothesised that this would change the solvent properties of the environment both to increase the loading and slow the release of free atRA. This was indeed what we showed, with increased amounts of API conjugate in the formulation allowing more free atRA to be dissolved in the oil. However, it was important to note that if too much was added the amount released was shown to be toxic in in vitro cell culture experiments. This gives the opportunity to tailor the amount of API conjugate added to achieve the appropriate amount of API released over an extended time frame. We demonstrated that the rate of release was independent of the amount of free atRA added (Table 3) and that with the addition of 10% of the API conjugate, we could extend the release of 80% of the dissolved atRA from 2 weeks in unmodified silicone oil up to around 50 days, which has been proposed as an appropriate time for treatment of PVR [20].

**Table 3:** Number of days taken to reach a certain cumulative percentage of atRA released from 5 and 10% (v/v) PDMS-atRA Blends with: (A) Saturated solutions of SIO1000; (B) Containing approximately the same initial amount of atRA, that is 50 µg mL^–1^ [19].

A			
Cumulative percentage	Days taken to reach percentage of release		
	SiO_1000_ (412.5 µg/mL)	5% Blend (700 µg/mL)	10% Blend (813 µg/mL)
**10**	<1	1.1	1.2
**20**	<1	1.8	1.8
**30**	1.6	2.9	3.1
**40**	2.4	4.1	4.2
**50**	3.3	6.8	7.2
**60**	5.4	9.2	9.9
**70**	8.2	12.8	16.4
**80**	**14.0**	**21.9**	**48.2**
**90**	35.0	–	–
**100**	–	–	–

B			
Cumulative percentage	Days taken to reach percentage of release		
	SiO_1000_ (49.2 µg/mL)	5% Blend (48.4 µg/mL)	10% Blend (46.2 µg/mL)
**10**	<1	<1	<1
**20**	<1	<1	<1
**30**	1.7	1.8	1.6
**40**	2.2	2.3	2.4
**50**	3.1	3.8	4.6
**60**	5.1	6.2	7.9
**70**	8.7	8.9	15.3
**80**	**16.2**	**17.1**	**50.8**
**90**	59.0	65.5	–
**100**	–	–	–

80%, the maximum cummulative percentage reached for all three oils, is highlighted in bold.

These studies demonstrate how exploitation of the chemistry of the silicone oil tamponade can modify the lipophilic environment. Using functional molecules able to interact with specific APIs, in combination with functional groups that promote solubility in the silicone oil, it is possible to tailor the release of the APIs over several weeks at therapeutic levels. At the same time, it is important to understand the influence of these additives on the physical properties of the tamponade agents to ensure that the viscosity and transparency are not adversely affected. We demonstrated that we could add relatively low concentrations of the additives while having a significant effect in prolonging delivery and reducing the likely influence on physical properties of the oils. Optimising these formulations can lead to new treatment strategies to enhance patient outcomes.

## Conclusion

The development of strong partnerships between materials scientists and clinical ophthalmologists can lead to opportunities to design new approaches to address sustained API delivery in the eye for the benefit of patients. It is essential to understand the specific therapeutic objectives and the limitations of current treatment regimens as well as what opportunities currently exist in terms of the use of medical devices and the materials from which they are manufactured. There has been a large amount of research in this area over many years although few have reached routine clinical practice. The ability to manipulate these materials and design new materials to promote the attachment and release of specific APIs in a targeted way could help to design particular solutions in different applications.
